# Robotic Adrenalectomy: A Portuguese National Health Service Experience

**DOI:** 10.7759/cureus.103202

**Published:** 2026-02-08

**Authors:** Mariana Mourao, Sofia C Guerreiro, Paula Tavares, Hugo Pinto Marques

**Affiliations:** 1 General Surgery, Hospital Curry Cabral, Unidade Local de Saúde São José, Lisbon, PRT

**Keywords:** adrenal glands, adrenal surgery, case-series, minimally invasive surgery, robotic adrenalectomy

## Abstract

Introduction: Minimally invasive adrenalectomy is currently the gold-standard approach for the surgical management of benign adrenal disease. In recent years, robotic-assisted adrenalectomy has gained increasing acceptance, offering technical advantages that may be particularly relevant in complex cases. The implementation of an endocrine robotic surgery program in a public hospital presents specific organizational and technical challenges.

Methods: We report a consecutive series of robotic transabdominal adrenalectomies performed at a tertiary, university-affiliated public hospital between April 2024 and December 2025. Demographic, perioperative, and postoperative outcomes were retrospectively analyzed.

Results: Thirty-two robotic adrenalectomies were performed (17 right-sided, 15 left-sided). The mean adrenal gland diameter was 6.2 cm, with a mean tumor diameter of 3.0 cm. Mean operative time was 110 minutes. Conversion to open surgery occurred in one patient (3%). Overall morbidity was 9.4%, including one pancreatic fistula and one incisional hernia requiring reoperation. There was no mortality. The mean hospital stay was 1.2 days.

Discussion: Our perioperative outcomes are comparable to those reported in the literature. Robotic adrenalectomy provides excellent visualization, enhanced dexterity, and ergonomic benefits, which may facilitate dissection in anatomically demanding situations. Emerging evidence suggests a potential role for robotic platforms in large, malignant, or technically complex adrenal tumors traditionally excluded from minimally invasive surgery.

Conclusion: Robotic adrenalectomy is a safe and feasible technique when implemented in specialized centers by experienced surgical teams. Its role may extend beyond benign disease to selected complex adrenal cases, although further studies are required to define its indications and cost-effectiveness.

## Introduction

Minimally invasive surgery is currently considered the gold standard for the treatment of benign adrenal disease. Since the first laparoscopic adrenalectomy described by Gagner et al. in 1992 [1], this approach has demonstrated clear benefits over open surgery, including reduced postoperative pain, shorter hospital stay, lower morbidity, faster recovery, and improved cosmetic outcomes [2].

The introduction of robotic-assisted surgery has further expanded the minimally invasive techniques available to endocrine surgeons. The first robotic adrenalectomy was reported in 2001 by Horgan and Vanuno [3], and since then, its adoption has steadily increased. Robotic platforms offer three-dimensional high-definition visualization, tremor filtration, articulated instruments with increased degrees of freedom, and improved surgeon ergonomics. These features are particularly advantageous in confined anatomical spaces such as the adrenal lodge.

Despite these technical advantages, comparative studies between robotic and laparoscopic adrenalectomy have not consistently demonstrated clear superiority of one approach over the other in standard cases [4]. Limitations of robotic surgery include increased costs, limited availability, and absence of haptic feedback. Nonetheless, robotic adrenalectomy may offer specific benefits in technically demanding procedures.

The present study aims to report the first Portuguese public healthcare system series of robotic transabdominal adrenalectomies, analyze perioperative outcomes, and review the current literature with particular emphasis on the role of robotics in adrenal surgery.

## Materials and methods

This retrospective observational study includes all consecutive patients undergoing robotic transabdominal adrenalectomy at Hospital Curry Cabral, a tertiary, university-affiliated center, between April 2024 and December 2025. The institution performs approximately 20-25 adrenalectomies per year.

Ethics approval

This study was designed as a retrospective, non-interventional analysis. Ethical approval was obtained from the Ethics Committee of Unidade Local de Saúde São José (Document No. I/1179/2026), as published in EXTRATO ATA No. RCA/2/2026.

Patient selection

Indications for surgery followed the European Society of Endocrinology guidelines for minimally invasive adrenalectomy [5]. All cases were discussed in a multidisciplinary endocrine board. Perioperative management was coordinated with the anesthesiology and endocrinology teams.

All procedures were performed using the da Vinci® Xi surgical system (Intuitive Surgical™, Mountain View, CA, USA) by the same experienced endocrine surgical team. The primary surgeon and assistant had extensive prior experience in laparoscopic adrenal surgery and formal robotic training. Initial procedures were proctored by an experienced robotic surgeon.

Surgical technique

**Figure 1:**
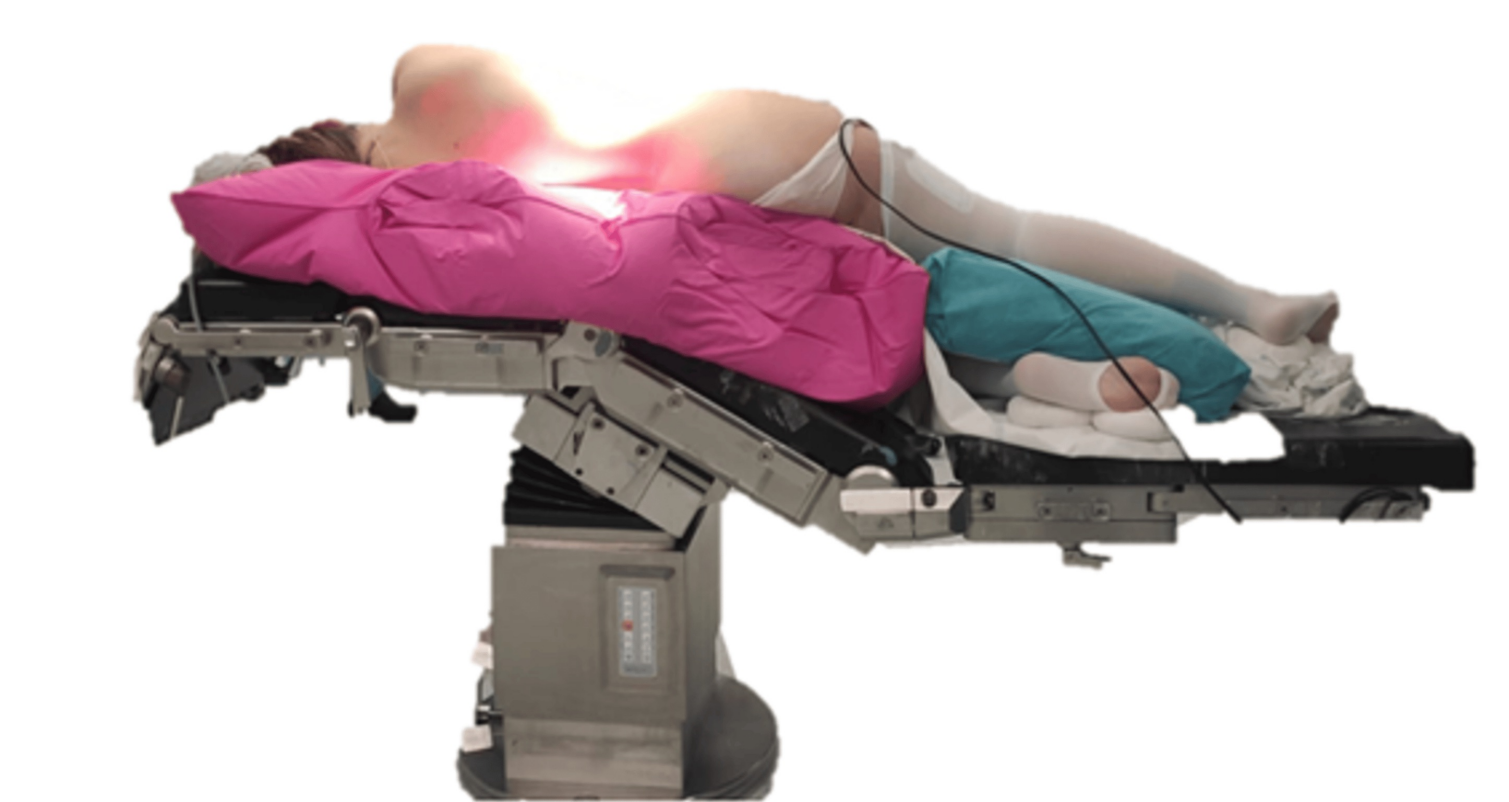
Photograph of patient's positioning for robotic adrenalectomy

The patient is positioned in a contralateral lateral decubitus position. A vacuum mattress is used to secure positioning and adequately protect pressure points. The operating table is flexed, and the lower limbs are slightly lowered to increase the operative workspace and optimize exposure of the retroperitoneum (Figure 1). Particular attention is paid to upper limb positioning to prevent brachial plexus injury.

All procedures are performed using the da Vinci® Xi robotic surgical system equipped with a 30° three-dimensional optical camera. Pneumoperitoneum is established using a standard technique, after which the trocars are placed. Ports are aligned in a horizontal configuration, with approximately 8 cm spacing between them to minimize the risk of robotic arm collision. The robotic platform is docked from the posterior aspect of the patient.

Dissection of the adrenal gland is carried out by the console surgeon using a robotic vessel-sealing device. The adrenal vein is carefully isolated and clipped by the bedside assistant. Following complete mobilization of the gland, the specimen is placed in an endoscopic retrieval bag and extracted through one of the port sites, which may be slightly enlarged if required. All 12-mm port sites are routinely closed under direct vision.

**Figure 2:**
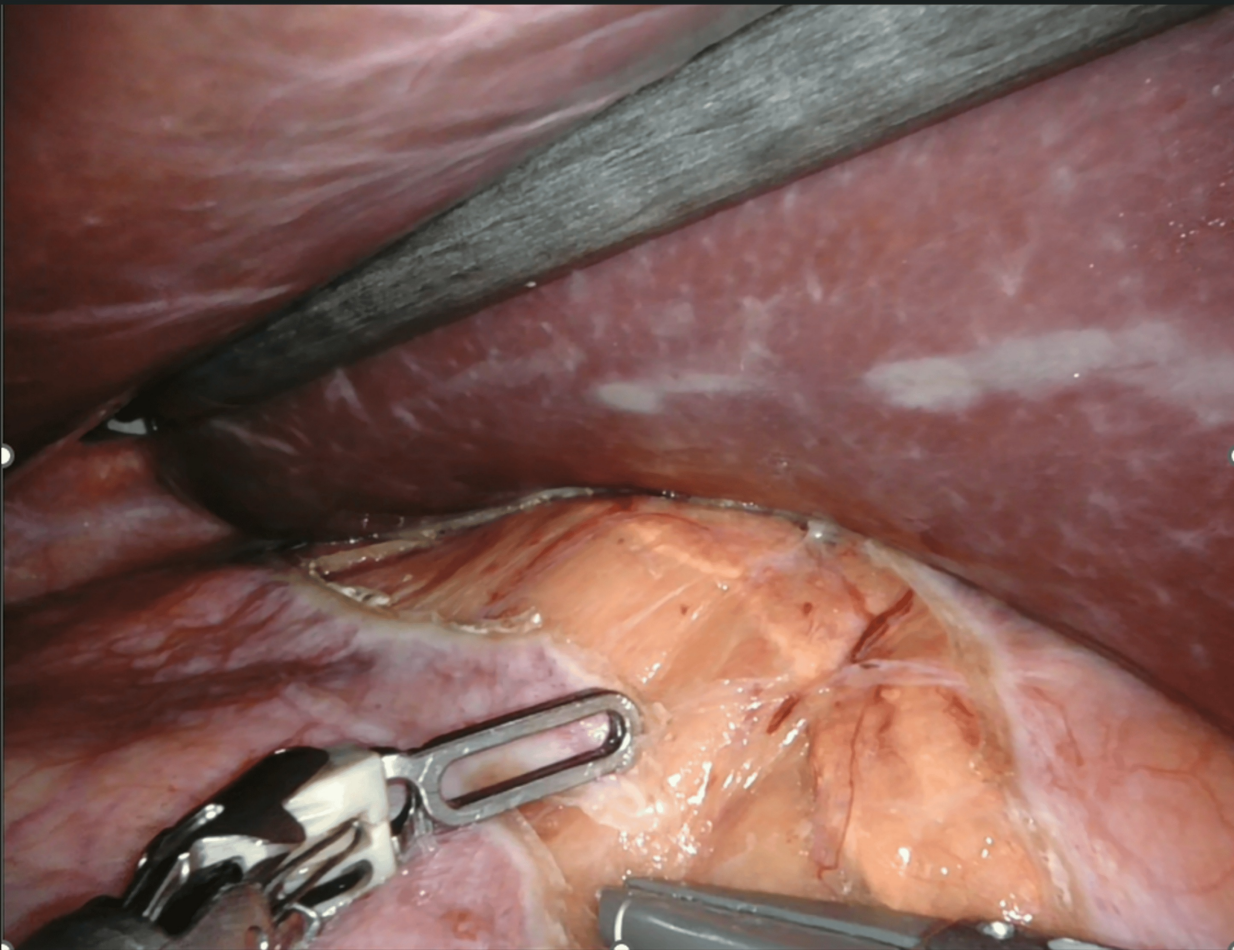
Right adrenalectomy: Hepatic flexure lowered and the liver retracted

**Figure 3:**
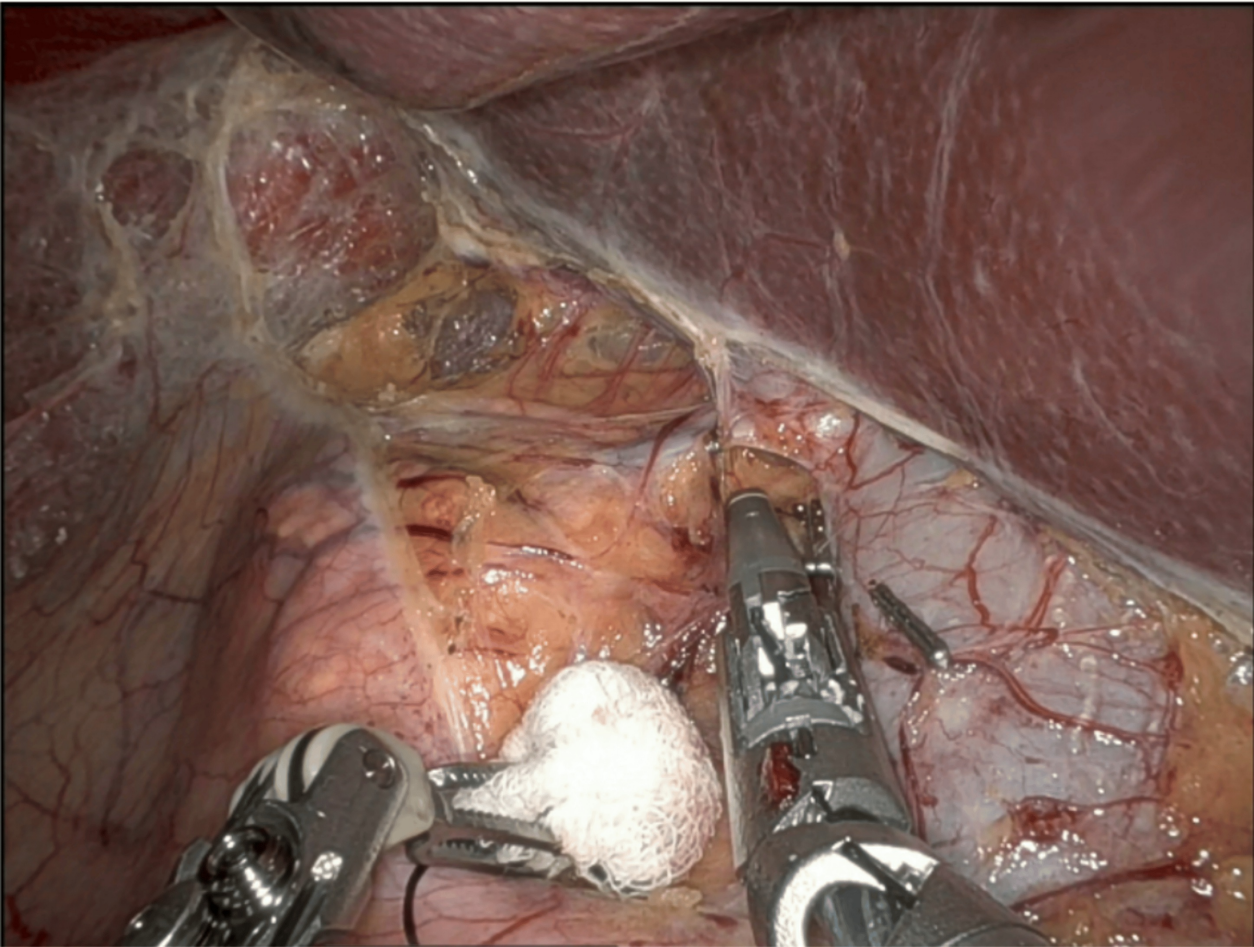
Right adrenalectomy: Right adrenal vein clipped

**Figure 4:**
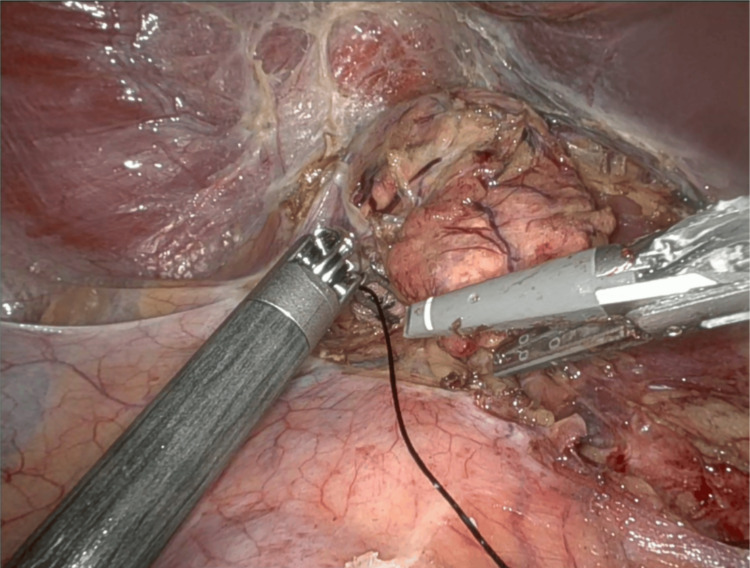
Right adrenalectomy: Remaining adrenal gland dissected

For right-sided robotic adrenalectomy, five ports are used: four robotic ports and one assistant port. The hepatic flexure of the colon is mobilized inferiorly, and the liver is retracted to allow adequate exposure of the right adrenal region (Figure 2). The peritoneum is incised to expose the inferior vena cava, and dissection proceeds in a medial-to-lateral fashion until the right adrenal vein is identified and clipped (Figure 3). The remaining attachments of the gland are then divided using the vessel-sealing device (Figure 4).

**Figure 5:**
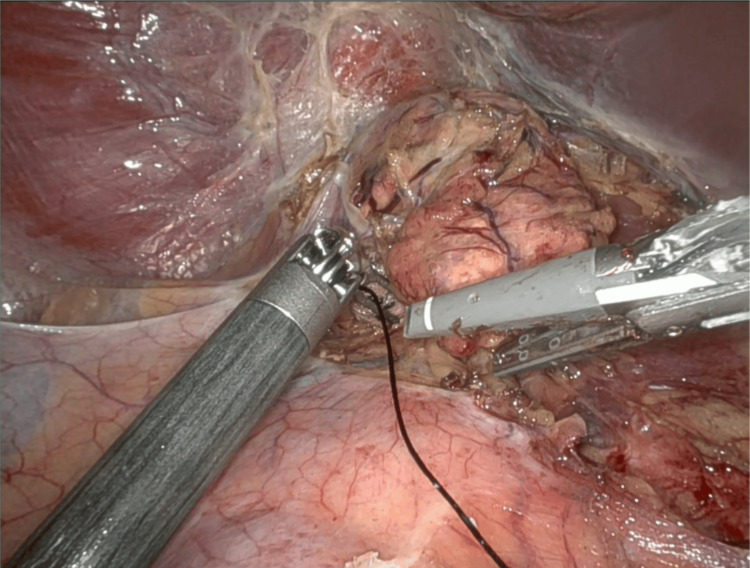
Left adrenalectomy: Splenic flexure lowered and adrenal exposure

**Figure 6:**
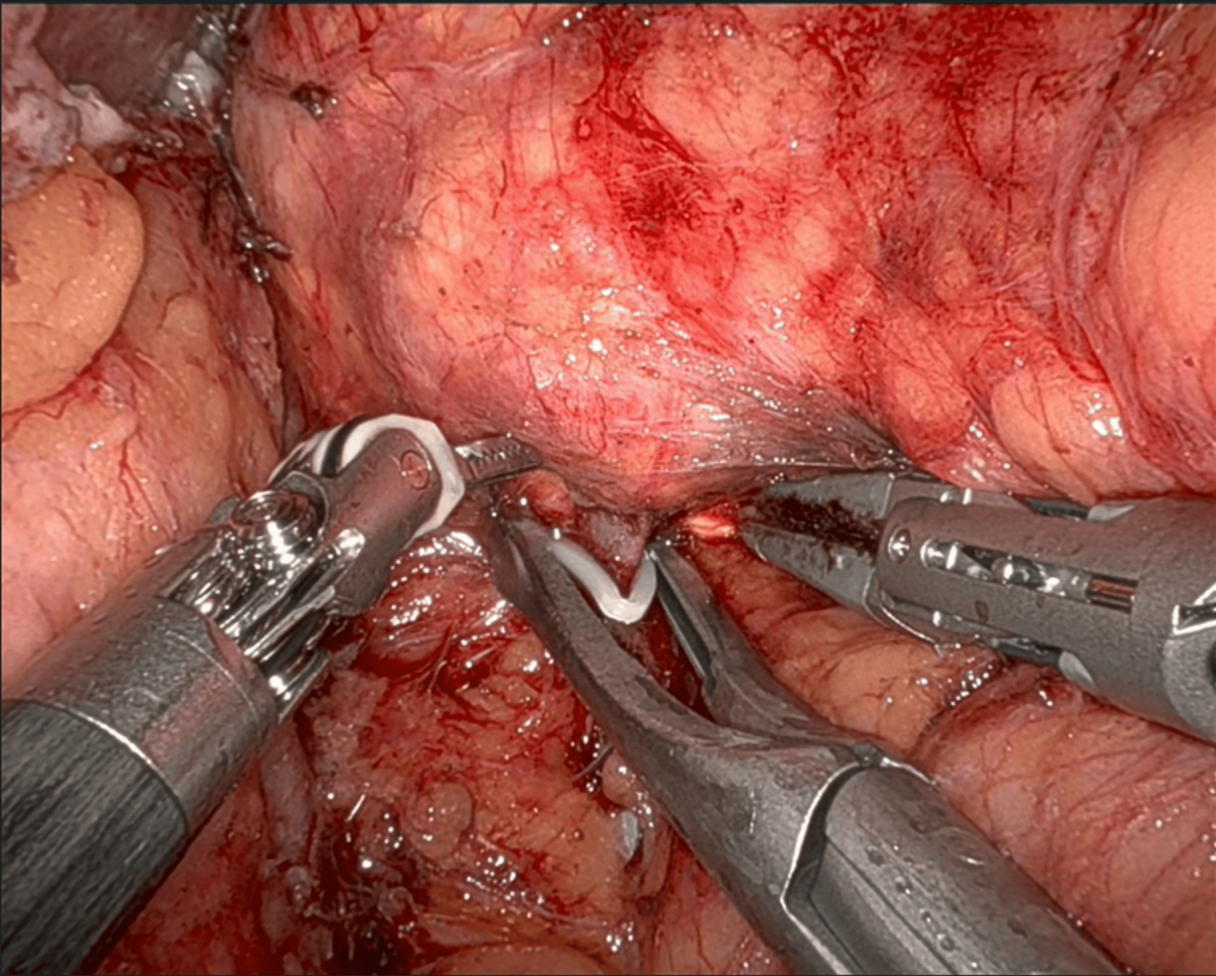
Left adrenalectomy: Left adrenal vein clipped

**Figure 7:**
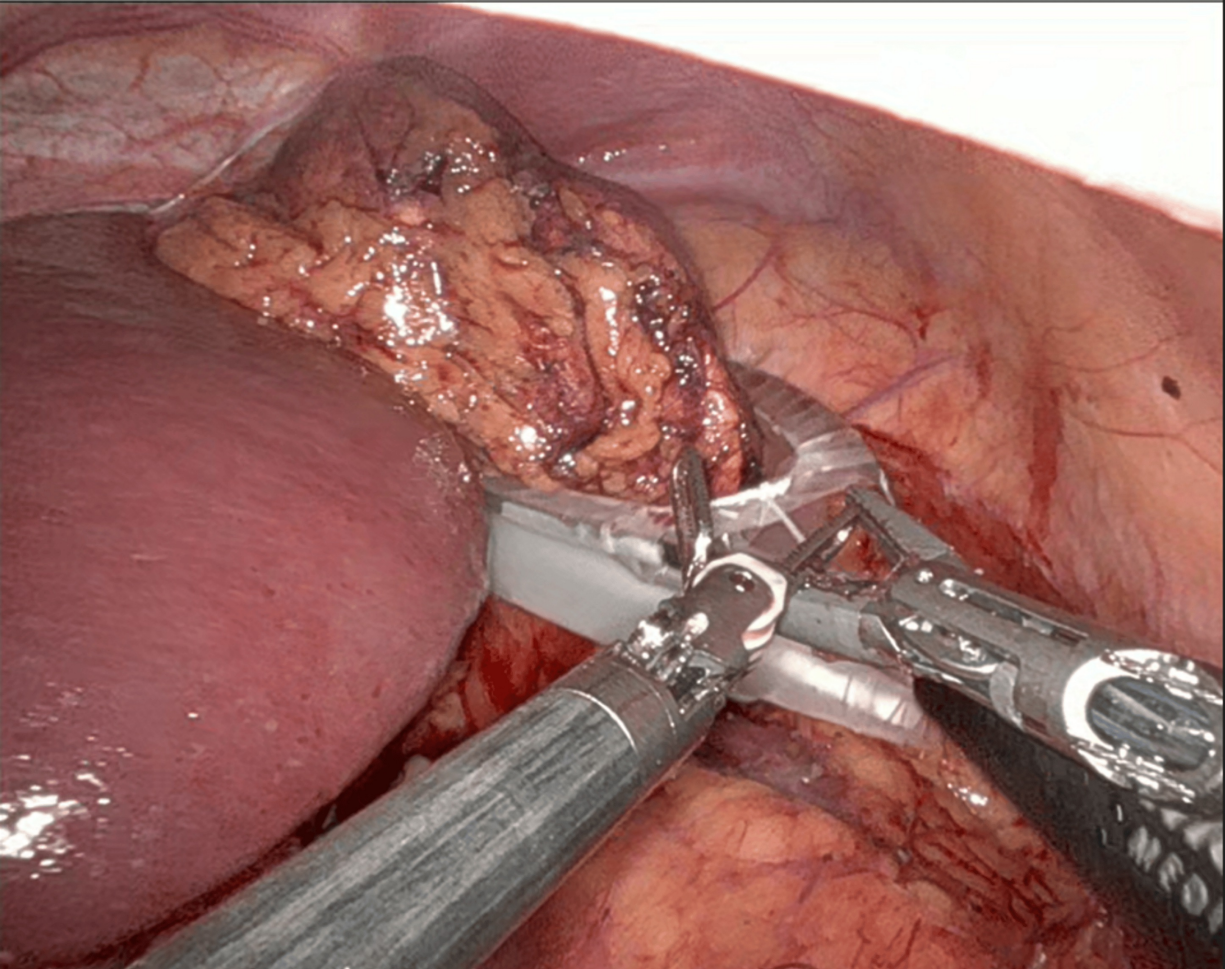
Left adrenalectomy: Gland dissection and extraction with endobag

For left-sided adrenalectomy, four ports are employed: three robotic ports and one assistant port. The splenic flexure of the colon is mobilized inferiorly to expose the left adrenal gland (Figure 5). The adrenal vein is carefully isolated and clipped (Figure 6), followed by complete dissection of the gland from the surrounding tissues (Figure 7). 

Routine placement of an abdominal drain is not performed.

Data collection and statistical analysis

Collected variables included demographic data, BMI (body mass index), ASA (American Society of Anesthesiologists) classification, indication for surgery, tumor size, operative time, conversion rate, length of stay, postoperative complications (Clavien-Dindo classification), histopathology, and mortality. Statistical analyses were performed using standard descriptive and regression methods, with continuous variables reported as mean ± standard deviation; significance was defined as a two-tailed p value < 0.05.

## Results

Thirty-two patients were included, with a female predominance (66%). Mean age was 55.2 years, and mean BMI was 29.3 kg/m². Most patients were ASA II-III. Primary hyperaldosteronism (68.8%) and Cushing syndrome (18.8%) were the most common indications (Table 1). The mean nodule diameter was 3.0 cm (Table 1).

**Table 1:** Patient's characteristics ASA: American Society of Anesthesiologists classification; BMI: Body mass index

Patient’s Characteristics		N=32
Gender	Female	21 (66%)
	Male	11 (34%)
Age	Mean age	55.2 years old
ASA	II	21 (66%)
	III	11 (34%)
BMI	Mean BMI	29.25 kg/m^2^
	<25	10 (31.2%)
	25-30	10 (31.2%)
	>30	12 (37.5%)
Pathology	Primary hyperaldosteronism	22 (68.8%)
	Cushing	6 (18.8%)
	Other	4 (12.5%)
Side	Right	17 (53%)
	Left	15 (47%)
Adrenal gland Diameter		6.2 (3 - 9)cm
Nodule Diameter		3.0 (1 – 9)cm

A total of 32 consecutive procedures performed by a single surgeon were included in the analysis. The mean operative time for the entire cohort was 109.5 ± 34.5 minutes.

When all procedures were analyzed together, a progressive reduction in operative time was observed across consecutive cases, demonstrating a pattern consistent with a learning curve. Linear regression analysis revealed a negative slope of -1.04 minutes per case, indicating increasing procedural efficiency with experience. Although this downward trend did not reach statistical significance (p = 0.12), it suggests a gradual improvement over time.

Seventeen procedures (53%) were performed on the right side. Operative time showed a decreasing trend with increasing case number. Linear regression demonstrated a negative slope of -1.58 minutes per case, consistent with a learning effect. This reduction did not reach statistical significance (p = 0.20).

**Figure 8:**
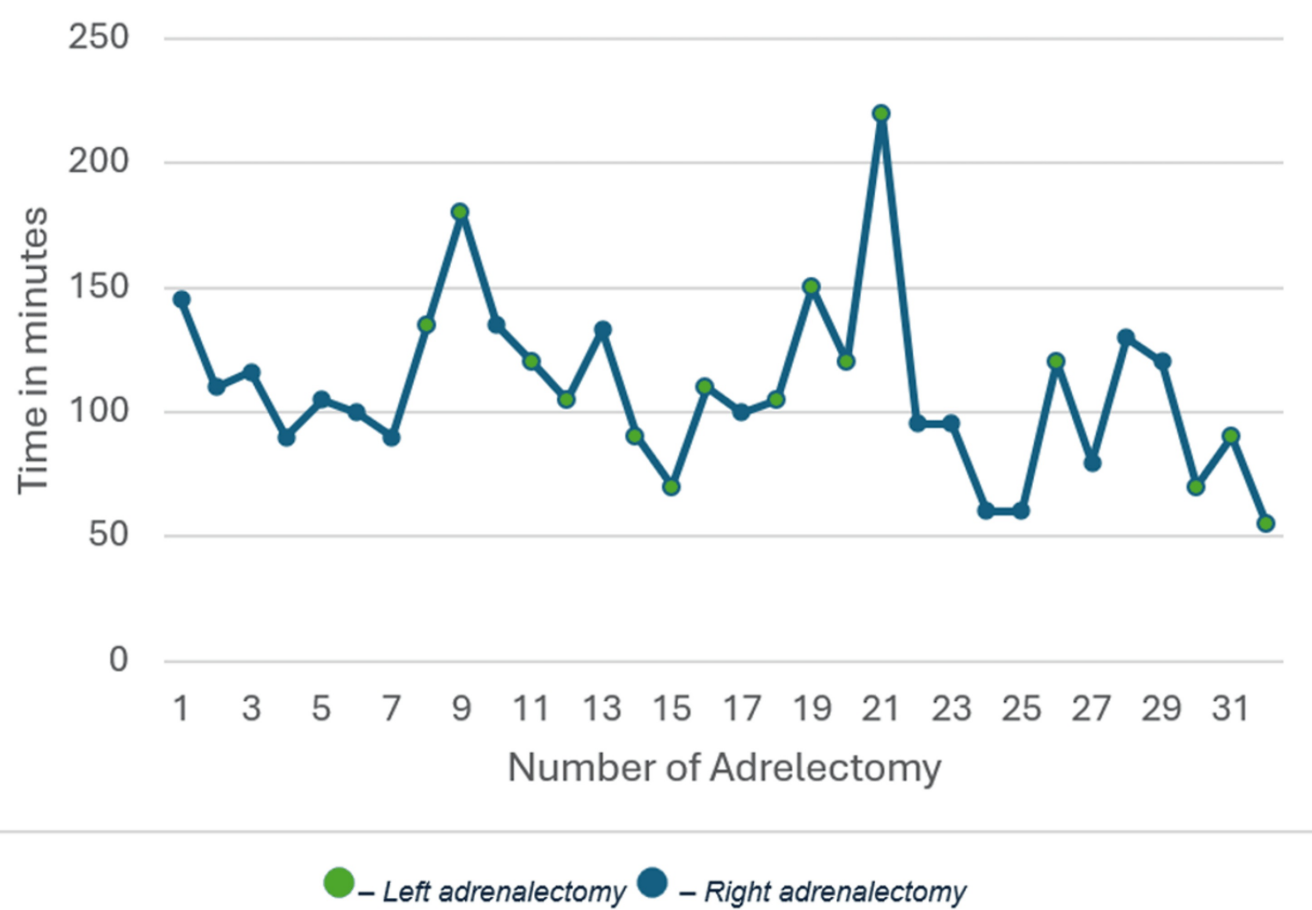
Evolution of overall intraoperative time

**Figure 9:**
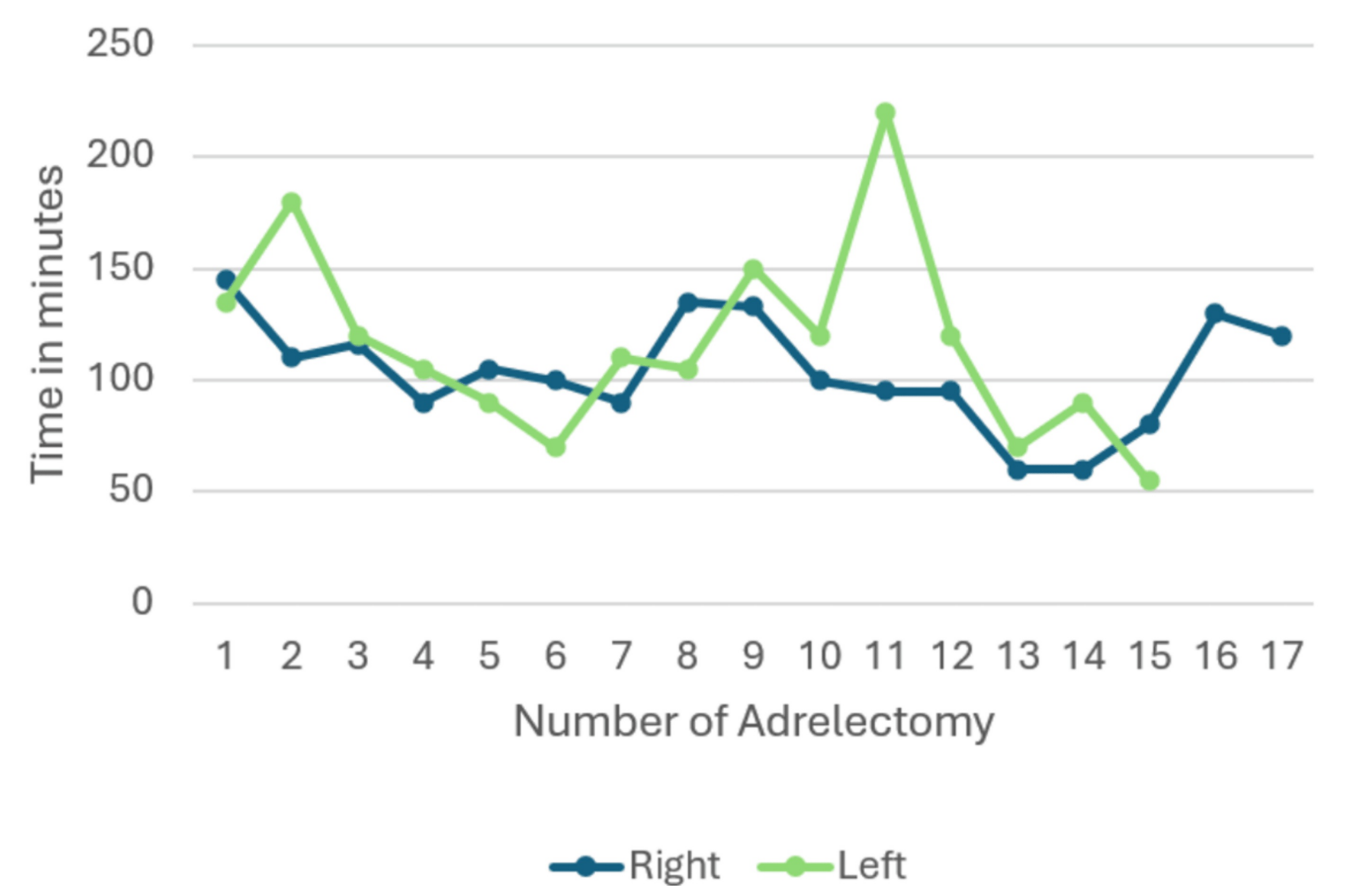
Evolution of intraoperative time for right and left adrenalectomy

Fifteen procedures (47%) were performed on the left side. A more pronounced reduction in operative time was observed compared to right-sided cases. Linear regression analysis revealed a negative slope of -2.71 minutes per case, indicating a steeper learning curve. However, this trend also did not reach statistical significance (p = 0.31), likely reflecting inter-case variability and limited sample size (Figures 8, 9).

The mean hospital length-of-stay was 1.2 days. One conversion to open surgery (3%) occurred due to pancreatic tail injury during left adrenalectomy. Overall morbidity was 9.4%, including two Clavien-Dindo II complications and one Clavien-Dindo IIIb complication. There was no perioperative mortality.

Histopathology revealed cortical adenoma in 84% and cortical hyperplasia in 10% of cases. The other cases were renal cell metastasis and myelolipoma (Table 2).

**Table 2:** Surgical outcomes

Outcomes	
Intraoperative time	
Overall	110 min
Right adrenal	103 min
Left adrenal	116 min
Conversion to laparotomy	1 (3%)
Mean Length of stay	1.2 days
Complications	
Total	3 (9.4%)
Right adrenal	-
Left adrenal	3 (20%)
Histopathology result	
Cortical adenoma	27 (84 %)
Cortical hyperplasia	3 (10%)
Others	2 (6%)

Postoperative morbidity cases

Case 1

One patient undergoing left robotic adrenalectomy required intraoperative conversion to an open subcostal left laparotomy due to an iatrogenic injury to the pancreatic tail. The injury was recognized during the procedure, and a surgical drain was placed at the end of the operation. In the postoperative period, the patient developed a pancreatic fistula, which was managed conservatively with antibiotic therapy and close clinical monitoring. The patient required hospital readmission but did not require further surgical or interventional procedures. The fistula resolved with conservative management, constituting a Clavien-Dindo grade II complication. This event occurred during the early phase of the learning curve, corresponding to the 9th robotic adrenalectomy overall and the 2nd left-sided procedure.

Case 2

Another patient developed an incisional hernia at one of the robotic port sites. The complication became clinically evident on postoperative day five, presenting with abdominal discomfort. The diagnosis was confirmed, and the patient underwent surgical repair under general anesthesia. This complication required reoperation and was therefore classified as a Clavien-Dindo grade IIIb complication. This case occurred during the 12th robotic adrenalectomy overall and the 4th left-sided adrenalectomy.

Case 3

A third patient presented to the emergency department five days after surgery with abdominal pain associated with a mild elevation of inflammatory markers. Abdominal computed tomography revealed a 4 cm intra-abdominal fluid collection located adjacent to one of the robotic port entry sites. The patient was admitted for intravenous antibiotic therapy and clinical observation. No invasive drainage was required. The clinical course was favorable, and the patient was discharged six days later. Follow-up computed tomography performed two weeks after surgery demonstrated complete resolution of the collection. This event was classified as a Clavien-Dindo grade II complication and occurred during the 30th robotic adrenalectomy overall and the 13th left-sided procedure.

## Discussion

This study represents the first reported Portuguese experience with robotic adrenalectomy. Our results demonstrate that the technique is safe and reproducible, with perioperative outcomes comparable to those reported by established international centers [6,7].

Robotic surgery was introduced into the Portuguese public healthcare system in 2019. By the end of 2023, a total of five robotic platforms were available nationwide. In 2024, a dedicated robotic endocrine surgery program was established at our institution, with adrenal surgery constituting its primary focus.

Given the relative rarity of adrenal pathology and the limited availability of robotic operating time, all procedures were centralized and performed by the same surgical team. Both the primary surgeon and the bedside assistant had extensive experience in endocrine surgery and laparoscopic adrenalectomy, in addition to formal training in robotic surgery. The initial four procedures, all right-sided adrenalectomies, were performed under the supervision of an experienced hepatobiliary robotic surgeon as part of a structured proctorship program.

The adrenal robotic surgery should be regarded in two phases: the patient positioning and docking phase and the console phase. Both should be considered in the learning curve and are important to reduce the overall intraoperative time.

Robotic adrenalectomy is associated with a relatively short learning curve, particularly for surgeons already experienced in laparoscopic adrenal surgery [8]. Our intraoperative times are comparable to those reported by other centers with a similar level of experience [9]. Our study demonstrates a trend toward reduced operative time with increasing experience, supporting the presence of a learning curve for this innovative surgical procedure. Although statistical significance was not reached, the consistent negative slopes observed in all analyses indicate progressive improvement in surgical efficiency over time.

Analyzing all procedures together was justified, as the same surgical technique was applied regardless of laterality. This global assessment reflects the overall acquisition of procedural proficiency by the surgeon. At the same time, stratified analysis by side provided additional insight into side-specific technical challenges.

Interestingly, left-sided procedures exhibited a steeper reduction in operative time compared to right-sided cases, suggesting a more pronounced learning effect. This may reflect greater initial technical complexity or anatomical constraints on the left side, leading to more substantial gains with experience. However, the absence of statistically significant differences between sides highlights the impact of limited sample size and variability inherent to early clinical experience with novel techniques.

The lack of statistical significance across models should be interpreted cautiously and does not negate the clinical relevance of the observed trends. Learning curve studies in surgery frequently demonstrate meaningful improvements that may not reach conventional thresholds of significance in small series. In this context, larger cohorts may help define when operative performance stabilizes and proficiency is achieved, a finding that in comparable surgical literature has been reported after an initial experience of approximately 10-20 robotic adrenalectomies [10,11].

Overall, these findings suggest that the procedure is associated with a progressive and continuous learning process, with measurable reductions in operative time as experience accumulates, and potential side-specific differences that warrant further investigation.

Our overall complication rate (9.4%) is within the range reported in the literature (9.2-12.5%) [12], and our conversion rate of 3% is comparable to that of other centers (2-13%) [12]. Incisional hernia is among the most frequently reported postoperative complications, with an incidence of approximately 2.6% [13]. In our series, this complication occurred in a patient with Cushing syndrome and required surgical repair. Pancreatic tail fistula is a rare but clinically relevant complication, particularly in left-sided adrenalectomy [13]. In our experience, it was managed conservatively with drainage and antibiotic therapy, with complete resolution.

Robotic adrenalectomy has been widely reported as a safe procedure with satisfactory perioperative outcomes; however, clear superiority over the laparoscopic approach in standard cases has not yet been established. Comparative studies have demonstrated similar operative times, length of hospital stay, and complication rates between the two techniques [14,15]. Nevertheless, robotic systems provide several technical advantages, including high-quality three-dimensional and stable visualization, enhanced instrument articulation with tremor filtration, and improved surgeon ergonomics [15,16]. Despite these benefits, the widespread adoption of robotic adrenalectomy remains limited by high costs and restricted availability, particularly within public healthcare systems.

Beyond routine cases, robotic surgery may offer distinct advantages in complex adrenal procedures. Large tumors suspected or confirmed malignancy, reoperative fields, obesity, and proximity to major vascular structures have traditionally been regarded as relative contraindications to minimally invasive surgery. In these challenging scenarios, the enhanced dexterity, precision, and visualization afforded by robotic platforms may facilitate safer and more controlled dissection. Several studies have demonstrated the feasibility of robotic adrenalectomy for large adrenal masses (>6-8 cm) and selected adrenocortical carcinomas without local invasion, reporting acceptable perioperative and early oncologic outcomes. Although long-term oncologic data remain limited, robotics may contribute to expanding minimally invasive indications in carefully selected patients when performed in high-volume specialized centers [17,18].

Given the relative rarity of adrenal pathology, centralization of care and performance by experienced, high-volume surgeons is essential to optimize outcomes, although the definition of a high-volume adrenal surgeon remains debated [19].

During the learning phase, structured proctorship and mentoring programs, together with the dissemination of institutional experience and outcomes, are essential for the safe implementation and development of robotic surgery programs worldwide [20].

This study has inherent limitations related to its retrospective, single-center design and relatively small sample size, which may limit statistical power and the generalizability of the findings. Although all procedures were performed by a single experienced surgical team, ensuring technical consistency, these results may not be directly extrapolated to other settings or surgeons. In addition, operative time was used as the primary surrogate for learning curve assessment and does not fully capture other aspects of surgical proficiency. Finally, the absence of a comparative laparoscopic group and cost-effectiveness analysis limits broader conclusions regarding the value of robotic adrenalectomy, highlighting the need for larger, prospective, multicenter studies.

## Conclusions

Robotic adrenalectomy is a safe and effective minimally invasive technique that can be successfully implemented in public healthcare systems. While its superiority over laparoscopy in standard cases remains unproven, robotics may play an important role in complex adrenal surgery, including large and selected malignant tumors. Centralization of care, surgeon experience, and structured training programs are essential for optimizing outcomes.
